# Is a 3 mm Surgical Margin Safe for Basal Cell Carcinoma in the Head and Neck that is Less than 2 cm, Considering Different Risk Factors?

**DOI:** 10.1055/a-2338-9192

**Published:** 2024-08-06

**Authors:** Min-Jun Yong, Seok-Ju Yoo, Hea-Kyeong Shin

**Affiliations:** 1 Department of Plastic and Reconstructive Surgery, Dongguk University College of Medicine, Gyeongju, Korea; 2 Department of Preventive Medicine, Dongguk University College of Medicine, Gyeongju, Korea

**Keywords:** skin cancer, excision margins, basal cell carcinoma, surgical flap, skin graft

## Abstract

**Background** Basal cell carcinoma (BCC) is the most common type of nonmelanoma skin cancer. Typically, resection requires a safety margin of ≥4 mm. When removing tumor cells, achieving complete excision with minimal safety margins and reconstructing the defect to preserve the original appearance are important. In this study, we used a 3-mm resection margin to confirm recurrence and re-resection rates.

**Methods** Electronic medical records and photographic data were obtained for patients with primary BCC lesions less than 2 cm in diameter who underwent wide excision with a 3-mm surgical margin from January 2015 to November 2021. We analyzed factors determining recurrence and re-resection rates, such as tumor size, location, age, sex, underlying diseases (including immunosuppression state), ethnicity, subtypes, tumor borders, etc.

**Results** This study included 205 patients. The mean age and follow-up period were 73.0 ± 11.5 years and 10.2 ± 8.0 months, respectively. The recurrence and re-resection rates were 1.95% and 25.85%, respectively.

A statistically significant correlation was found between recurrence rate and tumor border ( *p* = 0.013) and the re-resection rate was correlated statistically with location ( *p* = 0.022) and immunosuppressed patients ( *p* = 0.006).

**Conclusion** We found that a 3-mm excision margin provided sufficient safety in small facial BCC, resulting in ease of surgery and better aesthetic outcomes.

However, surgical margins must be determined case by case by integrating various patient factors. In particular, a surgical margin of ≥4 mm is required for BCC in high-risk areas, immunosuppressed patients, or poorly defined border.

## Introduction

Changes in society and medical care have led to a steady increase in the incidence of skin cancer. Basal cell carcinoma (BCC) is the most common nonmelanoma skin cancer. 1 2 3 The incidence of BCC increases each year, worldwide as well as in Korea. According to the Korean Statistical Information Service, BCC incidence rates have steadily increased more than double between 2011 and 2020, being 18.5 and 36.5 cases per 100,000 people, respectively, in these years. BCC development is influenced by several risk factors, including skin type and exposure to ultraviolet (UV) irradiation. Additional contributing factors include radiation treatment history, immunosuppression, arsenic exposure, scars, and hereditary disorders such as nevoid BCC syndrome (Gorlin–Goltz syndrome) and xeroderma pigmentosum. 4 5 Additionally, an increase in sun exposure over time results in an increased risk of developing BCC with age. Lesions ≥2 cm in diameter as well as those of morpheaform or infiltrative pathological subtype correspond to the high-risk BCC group. Continuously, high-risk factors include white skin with freckles or light hair, and poorly defined borders. On the other hand, lesions <2 cm in diameter, and pathological subtypes such as nodular or superficial or well-defined borders are classified as low-risk BCC group.

Immunosuppressed patients have an increased risk of skin cancer, and experience faster-growing cancers that are more likely to recur, metastasize, and cause death. 6 The National Comprehensive Cancer Network (NCCN) recognizes the increased risk of skin cancer recurrence in immunosuppressed patients, and recommends wide local excision or Mohs microsurgery, depending on the location and size, to treat all nonmelanoma skin cancers in these patients. 7 8

Nonsurgical treatments such as light amplification by stimulated emission of radiation ablation, medical management, immunotherapy, and chemotherapy are used for patients unable to undergo surgery. However, wide surgical resection with tumor-free margins is the preferred first line of treatment. This procedure includes the pathologist's confirmation for tumor-free on the peripheral margin of the resected tissue. Owing to this confirmation process, this procedure has a good curative rate, however, reconstruction of the defect is inevitably required. 9 Natural reconstruction of a large defect is generally more difficult. Often, a larger scar and donor site morbidity remain despite the defect being well-reconstructed. It is not good for aesthetic outcomes caused by sensory differences in texture, color, and even hypertrophic scars. To prevent unfavorable results, the surgical defect must be minimized and recovered with local flap coverage, rather than larger scale surgeries such as skin graft, regional, or free flap. 10

Aesthetically and functionally, the face is the most important part of the body, being particularly vital to a person's identity. Therefore, when removing tumor cells, it is important to achieve complete excision with minimal safety margins and preserve the original functional and aesthetic appearance while reconstructing the defect. However, optimal surgical margins for BCC remain controversial. The NCCN guidelines on surgical margin selection for BCC refer to as low- and high-risk lesions, based on the risk of recurrence. Low-risk BCC, defined as less than 2 cm in diameter, should undergo surgical excision using a 4-mm peripheral margin. The previous trend stated that a 4-mm safety margin was the most effective. However, for high-risk BCCs with a diameter greater than 2 cm, margins greater than 4 mm are recommended.

Based on several studies, 3 mm surgical margins were determined adequate to treat low-risk facial BCC. Therefore, we analyzed the factors determining recurrence and re-resection rates of small BCC resected with a 3-mm safety margin, including tumor size, location, age, sex, underlying diseases (including immunosuppression state), ethnicity, subtypes, tumor borders, etc.

## Methods

### Study Design and Patients

**Fig. 1:**
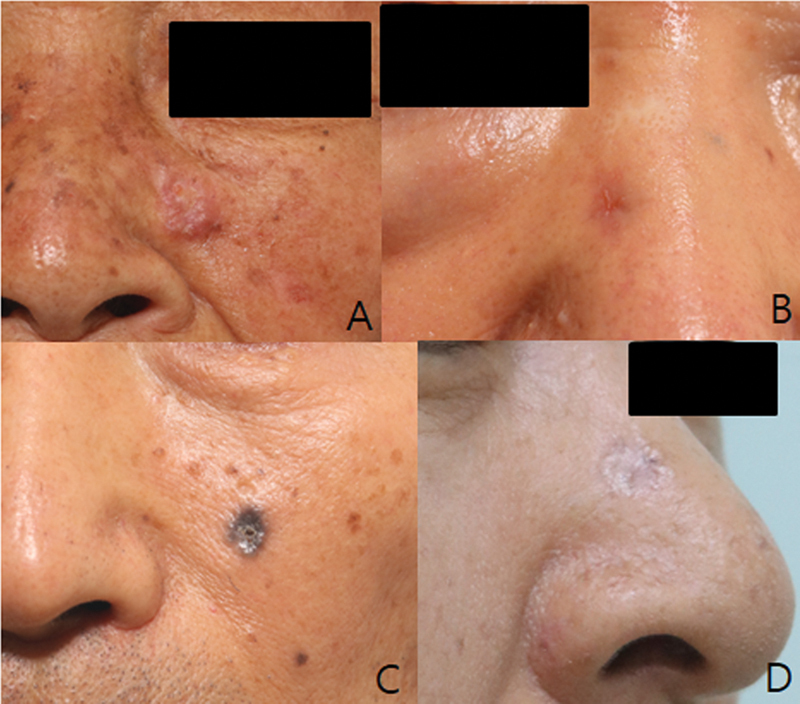
Clinical BCC subtypes: ( **A** ) Nodular, ( **B** ) superficial, ( **C** ) pigmented, and ( **D** ) morpheaform type. BCC, basal cell carcinoma.

This retrospective study included 205 patients with BCC of less than 2 cm in diameter who underwent surgery at our single center between January 2015 and November 2021. Electronic medical records (EMRs), including age, sex, tumor size, location, immunosuppressive status (due to underlying disease), and ethnicity were reviewed ( Table 1 ). All patients were classified into subtypes and tumor borders after visually identifying the lesions directly through photos taken and stored in advance. Based on the patient's clinical photo of the skin lesions, two doctors classified it into four subtypes using naked eye examination: nodular, superficial, pigmented, and morpheaform ( Fig. 1 ). They also categorized it based on whether it had a well-defined or poorly defined border.

**Table 1:** Epidemiological data of basal cell carcinoma

	Value ( *n* = 205)
**Age (years; mean ± SD)**	73.0 ± 11.5
**Sex**	
Male	82 (40.0%)
Female	123 (60.0%)
**Cancer size** **(cm; based on long axis)**	
<1	115 (56.1%)
1–2	90 (43.9%)
**Location**	
High-risk area a	161 (78.5%)
Low-risk area b	44 (21.5%)
**Operation method**	
Local flap	186 (90.7%)
Skin graft	18 (8.8%)
Primary repair	1 (0.5%)
**Immunosuppression drug**	
O	9 (4.4%)
X	196 (95.6%)
**F/U** **period (mean ± SD)**	10.2 ± 8.0
**Ethnicity**	
Asian	203 (99.0%)
White	2 (0.1%)
**Subtype**	
Nodular	146 (71.2%)
Superficial	45 (22.0%)
Pigmented	13 (6.3%)
Morpheaform	1 (0.5%)
**Tumor border**	
Well-defined	192 (93.7%)
Poorly defined	13 (6.3%)

Abbreviation: F/U, follow up; SD, standard deviation.

a High-risk areas: central face, eyebrows, periorbital area, nose, lips, chin, mandible, preauricular area, temple, and ears.

b Low-risk areas: cheeks, forehead, scalp, and neck.

### Surgical Technique

All patients underwent one or two surgeries for complete removal of the BCC. The inclusion criterion was the presence of BCC, while all cases of recurrence were excluded. BCC was resected using wide excision in all patients, with a pathologist confirming the absence of residual tumor on the resection margin. Considering the reconstruction that would follow, this procedure used, a safety margin of 3 mm, which is 1 mm less than the previous standard of 4 mm. All patients had an initial tumor resection margin of 3 mm. If the pathologist confirmed the presence of tumor cells at the resected margin, an additional resection of 1 mm was performed until no tumor cells remained on the frozen section biopsy.

A paper ruler was used to measure surgical defects caused by wide excision and additional excision. To cover defects, four plastic surgeons at our center performed a local flap, primary repair, or skin graft depending on the patient's condition, defect location, and size. Local flap consisted of advancement, transposition, and rotation flap. Direct closure can be performed through primary repair. The skin grafts included a split thickness skin graft and a full thickness skin graft using the anterior hairline, posterior auricle, or supraclaviclular area as donor sites. Even minimal defects required undermining of the peripheral flap margin and advancement due to lack of available skin and anatomic specificity.

### Recurrence Evaluation

All patients required regular revisits to our center for at least 6 months, to check for recurrence and operational scarring. When suspicious lesions or clinical symptoms such as color change, itching, redness, or wounds without a specific etiology were observed, a biopsy, followed by a pathological examination, was performed to confirm the recurrence of BCC.

### Statistical Analysis

Statistical analysis of categorical variables was conducted using the chi-square test. Nominal variables were analyzed using the chi-square test for normally distributed variables and the Fisher's exact test for non-normally distributed variables. Continuous variables were analyzed using the independent *t* -test for normally distributed variables and the Mann–Whitney U test for non-normally distributed variables. Data analysis was performed using *IBM SPSS Statistics ver. Using 27.0 for Windows* , univariate and multivariate analyses with major independent variables included were performed, and *p* < 0.05 was considered statistically significant.

## Results

A total of 205 patients, 82 male and 123 female, underwent surgery to remove BCC from their head and neck areas. The average age and follow-up period were 73.0 ± 11.5 years and 10.2 ± 8.0 months, respectively.

All patients had a tumor resection margin of 3 mm at the first resection. If no cancer cells were detected during simultaneous frozen section examination, reconstruction was performed. Surgical defects were covered using a local flap, skin graft, or primary repair. In this study, local flaps (186 cases) were most frequently used for defect coverage, followed by skin grafts (18 cases). Primary repair was only used in one case.

The recurrence and re-resection rates were measured after wide excision of the BCC with frozen sections and were found to be 1.95% (4 of 205) and 25.85% (53 of 205), respectively. Cancer cells were not found in the permanent specimen or frozen section.

There was no statistically significant correlation between recurrence rate and age, sex, cancer size, immunosuppressed state, location, ethnicity, or subtypes ( *p* > 0.05). However, a statistically significant correlation was found between recurrence rate and tumor borders ( *p* = 0.013; Table 2 ).

**Table 2:** Descriptive statistics for basal cell carcinoma recurrence and re-resection rates

	Number of patients		*p* -Value a			
			Univariate analysis		Multivariate analysis b	
	Recurrence rate	Re-resection rate	Recurrence rate	Re- resection rate	Recurrence rate	Re- resection rate
**Age (years)**			0.429	0.513	0.681	0.222
≥ 50	4	51				
<50	0	2				
**Sex**			1.000	0.794	0.233	0.865
M	2	22				
F	2	31				
** Cancer size (cm) c**			1.000	0.814	0.839	0.545
<1	2	29				
1–2	2	24				
**Location**			0.580	0.004	0.997	0.006
High-risk area d	4	49				
Low-risk area e	0	4				
**Operation method**			0.010	0.931	0.081	0.981
Local flap	2	48				
Skin graft	2	5				
Primary repair	0	0				
**Immunosuppressive drug** f			0.166	0.010	0.062	0.022
O	3	6				
X	1	47				
**Ethnicity**			1.000	0.451	1.000	0.177
Asian	4	52				
White	0	1				
**Subtype**			0.746	0.201	0.997	0.462
Nodular	4	38				
Superficial	0	14				
Pigmented	0	1				
Morpheaform	0	0				
**Tumor border**			0.021	0.745	0.013	0.922
Well-defined	2	49				
Poorly defined	2	4				

a Fisher's exact test and chi-square test. *p* < 0.05 is statistically significant.

b Multivariate analysis with age, sex, cancer size, location, operation method, and immunosuppressive drug.

c Cancer sizes are based on long axis.

d High-risk areas: central face, eyebrows, periorbital area, nose, lips, chin, mandible, preauricular area, temple, and ears.

e Low-risk areas: cheeks, forehead, scalp, and neck.

f Patients taking immunosuppressive drugs for breast cancer, chronic myeloleukemia, multiple myeloma, or kidney transplantation.

Re-resection rate did not correlate significantly with age, sex, cancer size, or follow-up duration ( *p* > 0.05). However, a statistically significant correlation was found between re-resection rate and immunosuppressed patients ( *p* = 0.022). Nine of the immunosuppressed patients regularly took immunosuppressive drugs for reasons including breast cancer, chronic myeloleukemia, multiple myeloma, or kidney transplantation.

**Fig. 2:**
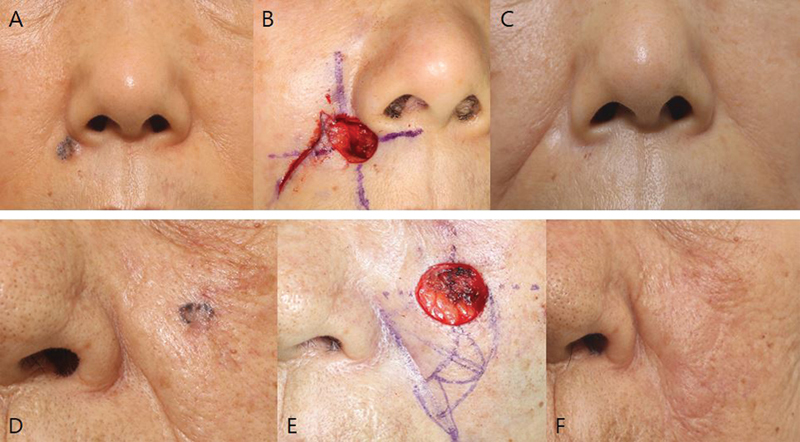
Patient photograph (high-risk area patient: [ **A–C** ], low-risk area patient [ **D** – **F** ]). ( **A** ) The BCC was confirmed in Rt. nasal alar groove area. Margins were demarcated according to gross appearance. ( **B** ) Wide excision was performed with a safety margin of 3 mm, but two additional re-resection were performed, and then reconstructed suing a rotation flap. ( **C** ) There was no recurrence at 24 months postoperatively, and the scar was not noticeable. ( **D** ) The BCC was confirmed on Lt. cheek. ( **E** ) Wide excision was performed with a safety margin of 3 mm and no additional resection was needed. ( **F** ) There was no recurrence at 12 months postoperatively, and the scar was not noticeable. BCC, basal cell carcinoma; Lt., left; Rt., right.

**Fig. 3:**
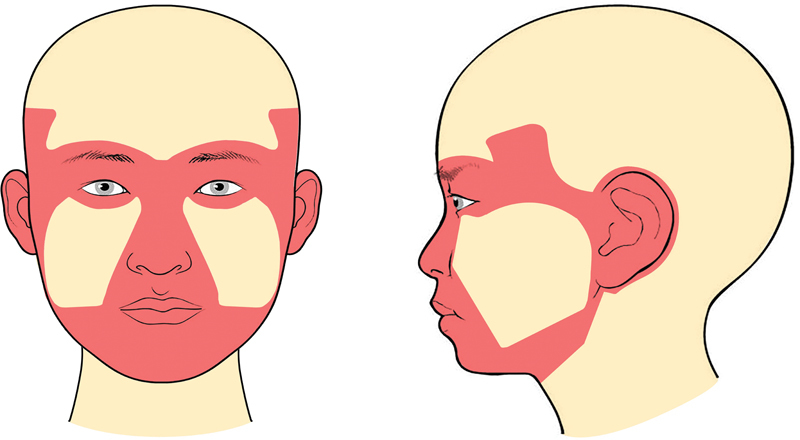
Schematic illustration. Location—high-risk areas (painted in red) and low-risk areas (painted in beige). The low-risk areas include cheeks, forehead, scalp, and neck, while the high-risk areas include what are commonly referred to as the “mask areas,” such as the central face, eyebrows, periorbital area, nose, lips, chin, mandible, preauricular area, temple, and ears.

In addition, when cancer location was divided into high- and low-risk areas, the re-resection rates were significantly lower for cancer in low- than in high-risk areas ( *p* = 0.006; Fig. 2 ). We divided the analysis into two risk areas: high and low. Low-risk areas include the cheeks, forehead, scalp, and neck, whereas high-risk areas are commonly referred to as the “mask areas,” such as the central face, eyebrows, periorbital area, nose, lips, chin, mandible, preauricular area, temple, and ears ( Fig. 3 ). 11

**Fig. 4:**
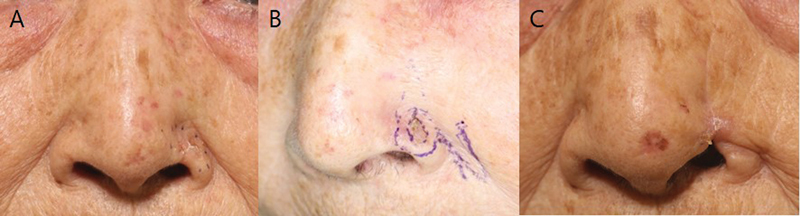
Patient photograph (recurrence patient, patient number 2). ( **A** ) The BCC was confirmed in Lt. nasal alar area. Margins were demarcated according to gross appearance. ( **B** ) Wide excision was performed with a safety margin of 3 mm in the initial surgery before recurrence. ( **C** ) BCC on nasal alar area was removed and local flap coverage was used to cover the defect. Recurrence was identified 48 months postoperatively. BCC, basal cell carcinoma; Lt., left.

Patients who experienced recurrence were reviewed at 24, 48, and 60 months ( Table 3 ; Fig. 4 ). In the cases of recurrence, wide excision was performed with a 6-mm resection margin.

**Table 3:** Details of patients with recurrent basal cell carcinoma

Patient details	Patient number			
	1	2	3	4
**Sex**	M	F	F	M
**Age**	76	74	63	61
** Cancer size (cm ^2^ ) **	0.5 × 1.2	0.7 × 1.0	0.4 × 0.5	0.3 × 0.3
**Location**	Nose High-risk area	Nose High-risk area	Medial canthus (a Rt. : Right) High-risk area	Lower lid (b Lt. : Left) High-risk area
**Reconstruction method**	c FTSG : Full-thickness skin graft	Local flap	FTSG	Local flap
**Recurrence interval (months)**	24	48	60	24
**Underlying disease**	–	d HTN : Hypertension, e CML : Chronic myeloleukemia	–	HTN
**Clinical/Pathological subtype**	Nodular	Nodular	Nodular	Nodular
**Tumor border**	Well-defined	Poorly defined	Poorly defined	Well-defined
**Follow-up period (months)**	48	60	68	8

Abbreviations: CML, chronic myeloleukemia; FTSG, full-thickness skin graft; HTN, hypertension; Lt., left; Rt., right.

## Discussion

BCC is the most common type of nonmelanoma skin cancer and, accounts for 77% of all skin cancers. 3 The incidence of BCC has increased worldwide over the last decade. 2 3 Nonmelanoma skin cancers are more frequent in older adults, reflecting the fact that cumulative sun exposure contributes to cancer development. 4 5

The prevalence of not only BCC but also other nonmelanoma skin cancers such as squamous cell carcinoma (SCC), dermatofibrosarcoma, and Merkel cell carcinoma is increasing. The surgical margins currently proposed are as follows: The NCCN guidelines on surgical margins refer to as low- and high-risk lesions, based on the risk of recurrence. Low-risk BCC should undergo surgical excision using a 4-mm peripheral margin. The previous trend stated that a 4-mm safety margin was the most effective. However, for high-risk BCCs, margins greater than 4 mm are recommended. The surgical margin recommended by the NCCN for the management of low-risk SCC is 4 to 6 mm. For high-risk SCC, margins greater than 6 mm are advised. The NCCN recommendation for the management of dermatofibrosarcoma protuberance is wide excision with 2- to 4-cm peripheral margins and deep margins extending to the investing fascia of the muscle or pericranium. 12 As with BCC, studies are being conducted to reduce surgical margins while performing wide excision in other nonmelanoma skin cancers.

Several recent studies have focused on reducing surgical margins and evaluating the possibility of excising small, well-defined primary BCCs using these reduced surgical margins. In a study conducted on 288 in Japan by Ito et al, in 2014, 218 patients (75.7%) underwent excision with a reduced margin (≤3 mm) and 60 patients (24.3%) had lesions excised with a wide margin (≥4 mm). The complete resection rates were 95.7% (44 of 46) in the ≤2 mm group and 100% (172 of 172) in the 3 mm group. Therefore, they concluded that the cure rate for 2- and 3-mm margins was 95.3% and 100%, respectively. However, these studies provided insufficient data for long-term outcomes. 13 In a study conducted by Lin et al in 2016, involving 143 patients, a 5-year follow-up design was employed to evaluate the adequacy of a 3-mm surgical margin for excision of both pigmented and nonpigmented BCCs. Recurrence served as the primary outcome measure. The study concluded that a 3-mm margin suffices for the excision of pigmented BCCs. However, nonpigmented BCCs exhibited a heightened risk of recurrence, warranting meticulous surveillance protocols and follow-up. 14 A similar investigation by Unverdi et al, in 2020, patients from 2016 to 2018 were scrutinized. The study encompassed 99 lesions from 91 patients diagnosed with BCCs ≤2 cm in size, subjected to excision with either 3 mm ( *n* = 53) or 5 mm ( *n* = 46) surgical margins. Among the lesions with 3-mm margins, only 3 out of 53 were margin positive-, while all 46 lesions with 5-mm margins were completely excised. Consequently, the study affirmed the safety and efficacy of utilizing a 3-mm margin for BCC excision. 15

Like other nonmelanoma skin cancers, BCC can be treated by complete resection, which inevitably requires wide excision. Complete resection of the BCC and Peripheral and Deep En Face Margin Assessment are the starting points for reconstruction, which can be problematic for large surgical defects. However, the anatomical characteristics of the facial area pose increased challenges to surgeons. Therefore, a 3-mm safety margin was trialed for small BCC to reduce the final defect. 16 Previous trends state that a 4-mm safety margin was the most effective in small BCC. Although the difference between 3 and 4 mm does not seem large, it is significant in small areas such as the medial canthus, eyelid, and nasal ala. Furthermore, even a small difference of a few millimeters in the face and neck region can lead to noticeable structural changes. We hypothesized that smaller resection margins in the facial region would not lead to high recurrence or re-resection rates and conducted research with 3 mm margins. While recurrence was confirmed in four cases, recurrence rates were generally comparable with those of previous studies. 17

Our analysis was divided into high- and low-risk areas. Low-risk areas include cheeks, forehead, scalp, and neck, whereas high-risk areas include what are commonly referred to as the “mask areas,” such as the central face, eyebrows, periorbital area, nose, lips, chin, mandible, preauricular area, temple, and ears. The re-resection rate was found to be significantly lower for cancer in the low-risk areas than for cancer in the high-risk areas. High-risk areas corresponding to the Mask area are often located in the more protruding parts of the face, such as the cheeks, nose, ear, and temples, which are typically exposed to high UVR levels. This can damage skin cells, leading to DNA damage, and increase the risk of BCC development. Therefore, it is important to distinguish between high- and low-risk areas for BCC development and plan surgery with appropriate surgical margins for each location. Through this approach, all tumors can be removed with appropriate surgery, while preventing possible recurrence or re-resection.

In addition, this study confirmed that re-resection rates were statistically significantly higher in immunosuppressed patients. Ten of the immunosuppressed patients regularly took immunosuppressive drugs for breast cancer, chronic myeloleukemia, multiple myeloma, or kidney transplantation. Immunosuppressed patients are at markedly increased risk of developing cutaneous malignancies compared with the general population. 6 In cancer, regulatory T cells are recruited as a subpopulation of tumor-infiltrating lymphocytes, whose accumulation at tumor sites is thought to impede T-cell immunity to tumor-associated antigens. 7 8 Regulatory T cells play a role in forming an immunosuppressed niche in the facial skin, which may have pathogenic consequences for the development of skin cancer. 18 Therefore, skin cancer in immunosuppressed patients may exhibit higher rates of wider and deeper spread, leading to higher recurrence rates.

And, our study confirmed that a statistically significant correlation was found between recurrence rate and poorly defined tumor border. In instances characterized by clinically unclear tumor borders, lesions may manifest as flat, ill-defined skin areas exhibiting subtle alterations in texture and coloration.

In cases where tumor borders are ambiguous, it is harder for pathologists to accurately assess whether the surgical margins (the area of tissue surrounding the tumor that has been removed) are free of cancer cells. The “spread out” nature of malignant cells amidst interspersed normal-appearing tissue heightens the probability of histopathologists categorizing excision lesion margins as clear, potentially overlooking residual disease. 19 If there are cancer cells present at the margins, it increases the likelihood of tumor recurrence.

This study had some limitations. First, this was a retrospective study reviewing EMRs and photographic data and the small sample size was not sufficient to obtain statistically meaningful results. Second, due to the retrospective study, therefore, we did not include a control group. Third, continuous follow-up was not feasible for all patients due to factors such as low compliance and death due to old age, making statistical analysis difficult. Fourth, as this was a single-institution study, there may have been bias. Fifth, recurrence rates vary depending on the histological cancer type; however, this cannot be confirmed during surgery. Consequently, the operator has to use the gross appearance of the tumor to determine the extent of resection required. Lastly, dermoscopy is nowadays an integrative part of the clinical examination and in the management of patients with skin tumors. 20 Our study is retrospective in nature, and dermoscopy was not universally utilized among all patients. Given our hospital's protocols, the majority of patients suspected of skin cancer typically initiated their medical journey by consulting a dermatologist, undergoing a punch biopsy as an initial diagnostic step, and subsequently receiving a pathology department diagnosis before returning to the hospital for further management. Therefore, the application of dermoscopy for border detection was not feasible during the initial patient visits to the hospital in the majority of cases. Nonetheless, conveying the precise tumor border remains crucial for ensuring thorough removal. Therefore, we visually examined patient photographs and classified tumor borders for skin lesions to investigate any potential correlation with recurrence or re-resection rates. Statistical significance between recurrence and tumor border was confirmed. Consequently, even in cases where dermoscopy is unavailable, visual assessment of the tumor border is imperative. It allows for individualized surgical margin delineation, thus facilitating surgical planning. However, as previously stated, patients have often undergone punch biopsies prior to hospital visits, and classification based on naked eye examination of photographs has been employed. Thus, additional research is warranted to validate these findings using dermoscopy.

Many plastic surgeons have focused on appropriate surgical safety margins and surrounding tissues in order to completely resect BCC. Using a 3-mm surgical resection margin rather than a wider one makes it easier for the surgeon to reconstruct the facial defect after wide excision. Therefore, performing 3-mm resection in the facial region based on clinical appearance is comparable to previously proposed methods as defect size was reduced without altering the recurrence rate. Considering the difficulty of reconstructing the facial area, a 3-mm resection margin is recommended for wide excision of BCC. However, it is important to distinguish between high- and low-risk areas for BCC development and plan for surgery with appropriate surgical margins for each location.

In immunosuppressed patients, skin cancer may exhibit higher rates with wider and deeper spread, leading to higher recurrence rates. Therefore, it is necessary to comprehensively understand the disease pathogenesis, so that optimal management strategies, including surgical planning with appropriate margins, can be developed to ensure the best outcomes for these patients.

In instances of tumors exhibiting poorly defined tumor borders, discerning between normal and cancerous tissues poses a challenge, necessitating heightened vigilance and meticulous consideration. The authors advocated for resecting BCCs with unclear margins utilizing wider peripheral and deeper margins, aiming to thoroughly eradicate any asymptomatic tumor spread. Additionally, they suggested maintaining a lower threshold for reexcision, contingent upon the pathological assessment of resection margins.

As a result, it is necessary to set the surgical margin by integrating various patient factors. In this study, we conclude that performing 3-mm resection in the head and neck region of small BCC based on their clinical appearance is comparable with previously used methods, as the defect can be narrowed without altering the recurrence rate and re-resection rate. However, a surgical margin of ≥4 mm is required for small BCC in high-risk areas, immunosuppressed patients, and poorly defined tumor border.
